# Corticosteroid use for 2019-nCoV infection: A double-edged sword

**DOI:** 10.1017/ice.2020.165

**Published:** 2020-04-23

**Authors:** Sundus Nasim, Sohail Kumar, Dua Azim, Zehra Ashraf, Qiraat Azeem

**Affiliations:** 1 MBBS, Dow Medical College, Dow University of Health Sciences, Karachi, Pakistan; 2 Faculty of Pharmacy, Jinnah University for Women, Karachi, Pakistan

*To the Editor—*A newly emergent coronavirus disease 2019 (COVID-19), first recognized in the city of Wuhan, China, in early December 2019, is a respiratory tract infection. On March 11, 2020, with >118,000 cases reported in 114 countries and nearly 4,291 deaths worldwide, this virus was labeled a pandemic by the World Health Organization (WHO).^1^ Responding to the uncertain clinical progression of COVID-19 and the absence of any particular therapy with established efficacy, the medical and scientific communities are working to develop various therapies to achieve an effective cure for the COVID-19. Some physicians have suggested corticosteroids to treat COVID-19 as in previous outbreaks of severe acute respiratory syndrome (SARS) and the Middle East respiratory syndrome (MERS).

Although corticosteroid use has been reported in hospitalized patients with severe disease, contradictory evidence from the WHO regarding corticosteroid use in some viral illnesses suggests that this evidence is not definitive. In severe cases of COVID-19, complications (eg, pneumonia, acute respiratory distress syndrome, cardiomyopathy, arrhythmia, acute kidney failure, sepsis, and septic shock) can occur along with complications associated with prolonged hospitalization (eg, secondary bacterial infections). In severely ill patients with these complications, corticosteroids have been used widely.^2-4^ During a retrospective review in Wuhan Union Hospital,^5^ the efficacy of the early use of short-term corticosteroids was investigated and compared with a control group using the clinical record and chest computed tomography (CT) scans. Among these groups, one group was intravenously administered methylprednisolone at a dose of 1–2 mg/kg/d for 5–7 days. The results included the rapid return of body temperature to a normal and improvement in peripheral capillary oxygen saturation (SpO_2_). Chest CTs showed improved absorption focus with methylprednisolone administration. Parallel to these reported observations, another Chinese study showed similar outcomes with early use of high-dose corticosteroids along with quinolone in patients with severe acute respiratory syndrome coronavirus (SARS-CoV).^6^

Contrary to the aforementioned results, the current interim guidance from WHO on clinical management of severe acute respiratory infection when COVID-19 is suspected (released January 28, 2020),^7^ advises against the utilization of corticosteroids during this disease unless it is indicated for a comorbid clinical condition. The wide use of this drug in the management of SARS-CoV and the Middle East respiratory syndrome coronavirus (MERS-CoV) worsened the immune response and caused diffuse alveolar damage, even though it did suppress lung inflammation to some extent.^8,9^ In a review of observational studies on SARS patients with progressively worsening pulmonary conditions or abnormalities on chest X-ray, those administered corticosteroids showed no benefit but did show possible side effects such as steroid-induced psychosis and avascular necrosis. This review classified the treatment regimens as early treatments and rescue treatments administered in later stages of the disease progression.^10^ Russell et al^11^ summarized the results of various case-control studies of SARS patients; they showed a higher incidence of psychosis with high-doses of corticosteroid administration, as well as diabetes, delayed viral clearance, and avascular necrosis. They also reported a delay in viral RNA clearance from the respiratory tract following corticosteroid administration in a MERS-CoV infection.^11^

Considering these findings, no evidence exists to indicate that the use of corticosteroids will benefit patients infected with 2019-nCoV, and it could worsen their condition. In conclusion, we understand that the ongoing coronavirus pandemic is a challenging and unprecedented time for the world. Although few studies do suggest a potential role for the use of corticosteroids in COVID-19 treatment, the current literature does not provide any definitive evidence for or against their use. Thus the use of corticosteroid could be regarded as a double-edged sword. Corticosteroid treatment ought not to be utilized for the treatment of COVID-19 outside of a clinical trial, and caution should be exercised until further evidence regarding corticosteroid use specific to COVID-19 emerges. However, we recommend that clinicians proceed with extreme caution when administering corticosteroids, making it certain that the benefits outweigh the risks.
